# Septin4 promotes cell death in human colon cancer cells by interacting with BAX

**DOI:** 10.7150/ijbs.44429

**Published:** 2020-04-07

**Authors:** Xin Zhao, Hao Feng, Yang Wang, Yanmei Wu, Qiqiang Guo, Yanling Feng, Mengtao Ma, Wendong Guo, Xiaoyu Song, Ying Zhang, Shuai Han, Liu Cao

**Affiliations:** 1 Key Laboratory of Medical Cell Biology, Ministry of Education; Institute of Translational Medicine, Collegeof Medical Science, China Medical University; Liaoning Province, Collaborative Innovation Center of Aging Related Disease Diagnosis and Treatment and Prevention, Shenyang, Liaoning Province, China; 2 Department of Ophthalmology, The First Hospital of China Medical University, Shenyang, Liaoning Province, China; 3 Panjin Liaohe Oilfield Gem Flower Hospital, Panjin, Liaoning Province, China; 4 Department of Cardiology, the First Hospital of China Medical University, Shenyang, Liaoning, China.; 5 Department of Neurosurgery, the First Hospital of China Medical University, Shenyang Liaoning Province, China

**Keywords:** Septin4, BAX, Colon Cancer, apoptosis

## Abstract

Septin4 is a tumor suppressor protein that promotes cell programmed death in various cell types through specifically antagonizing XIAP (X linked inhibitor of apoptosis), little is known its other novel binding partner and role in colorectal cancer. In this study, we found that Septin4 significantly expressed lower in human colon cancer when compared to peri-tumor benign cells, and its low expression was significantly associated with worse prognostic outcomes. Furthermore, Septin4 participated in DOX-induced colon cancer cell death* in vitro*. Septin4-overexpressing colon cancer cells displayed augmented apoptotic cell death and ROS production. Additionally, Septin4-knockdown cells revealed a resistance of DOX-induced cell death and reduced ROS production. Importantly, we first identified that BAX is a novel Septin4 binding partner and the interaction is enhanced under DOX treatment. Finally, Septin4-knockdown promoted colon cells growth *in vivo*. These observations suggest that Septin4 as an essential molecule contribute to the occurrence and development of human colon cancer and provide new technical approaches for targeted treatment of this disease.

## Introduction

Colorectal cancer (CRC) is the third most common malignant tumor in the world after lung cancer and gastric cancer and is also the second most lethal tumor 1. Though potential risk factors can be changed, somatic gene mutations and epigenetic abnormalities can induce tumor occurrence 2. In the past few decades, our understanding of the epidemiology, etiology, molecular biology, and clinical aspects of CRC has greatly advanced 3. Nevertheless, 1.8 million new CRC cases are reported every year worldwide. Diagnosis is usually made in the later clinical stages of this disease, and thus, approximately 900,000 people die of this malignancy each year. In relatively underdeveloped countries, the existing screening methods and available medical means are not enough to halt the rising trend of these rates 3. Therefore, to prevent the occurrence and development of CRC, and to provide effective strategies for clinical treatment, it is essential to understand the pathogenesis and the molecular mechanism of this disease.

Apoptosis has critical roles in organ development, tissue homeostasis, and defense mechanisms that can remove useless or potentially cancerous cells 4, 5. Decreased apoptosis can lead to a variety of pathological processes, including cancer 6, and caspase activation is the ultimate marker of apoptosis. Because their activation can lead to a series of serious consequences related to apoptosis, caspases are precisely regulated by different positive or negative regulatory factors 7. In living cells, X-linked inhibitor of apoptosis (XIAP) can directly bind to caspases, inhibit caspase activity, and negatively regulate apoptosis 8. Therefore, XIAP antagonistic proteins are necessary to promote cell death and maintain tissue renewal by inhibiting XIAP activity 9-11.

Septin4 is the most important XIAP antagonist proteins, located in the outer mitochondrial membrane 11-14. When apoptosis occurs, spetin4 can translocate from the mitochondria to cytoplasm and directly bind to XIAP, thus activating caspases and leading to cell death 15, 16. Moreover, unlike traditional tumor suppressors or oncogenes, whose loss- or gain-of-function mutations can lead to the occurrence of cancer 17, Septin4 can affect tumor occurrence through expression changes, which makes it a more universal cancer suppressor. Studies have shown that the expression of Septin4 is decreased in acute lymphoblastic leukemia, and the proportion of hematopoietic stem cells can be increased through resistance to apoptosis by knocking out Septin4^18^, ^19^. Recent studies further demonstrated that one of the major proteins encoded by the Septin4 gene, Sept4_i2/ARTS, is highly expressed in the intestinal stem cells (ISCs) in the niche of the crypts of Lieberkuhn. Sept4_i2/ARTS can regulate the number of ISCs in the intestinal crypt by promoting apoptosis to maintain the homeostasis and regeneration of intestinal epithelial cells 20. With the recent in-depth study of the cell biological function of Septin4, accumulating evidence has suggested that Septin4 is involved in the regulation of tumor cell death, proliferation, angiogenesis, and invasion, which is a marker of these molecular mechanisms 21.

The mitochondrial apoptosis pathway is regulated by Bcl-2 family members, maintaining the basic balance of cell survival and death 22. Breaking this balance can lead to pathological proliferation of cells, such as cancer or autoimmune diseases and pathological cell death, including neurodegenerative diseases or bone marrow failure. Mitochondrial outer membrane permeability (MOMP) marks the outcome of Bcl2-family-regulated apoptosis 23. BAX, an apoptosis-promoting protein with multiple BH domains, is located in the outer membrane of mitochondria and has the ability to self-assemble into homologous oligomers to decrease MOMP 24. BAX exists in almost all cell types, including tumor cells. The early model of apoptosis activation suggested that BAX activation is regulated by the inhibitory interaction of anti-apoptotic members on the outer mitochondrial membrane 24. Therefore, to activate apoptosis, BAX needs to be replaced and activated by the anti-apoptotic protein complex to form homologous oligomers and trigger MOMP.

In this study, we first confirmed that Septin4 expression levels were decreased with increased colon cancer grade, and patients with high expression of Septin4 had a good prognosis. We confirmed that Septin4 can promote the apoptosis of colon cancer cells by binding to BAX. And Septin4 knockdown accelerated subcutaneous tumor growth *in vivo.* These findings may contribute to future research on the occurrence and development of CRC and provide new technical approaches for targeted treatment of CRC.

## Materials and Methods

### Cell culture

Human colon cancer cells (HCT116) were purchased from the American Type Culture Collection (ATCC, USA) and cultured in 1640 medium (HyClone, GE lifescience, UK) supplemented with 10% fetal bovine serum (FBS; Clark Bioscience, Richmond, VA), penicillin (100 units/ml), and streptomycin (100 μg/ml). All cells were cultured in a humidified incubator at 37 °C with 5% (v/v) CO_2_.

### Liposome transfection

The eukaryotic expression plasmid GV141-Septin4-3×Flag was purchased from Genechem Co.,Ltd. (Shanghai, China) The cells were seeded into 6 cm plates one day before transfection, with the confluence of the transfected cells reaching 70%-80% the next day. Cells were transfected with plasmids using Lipofectamine 3000 (Invitrogen, USA) according to the manufacturer's instructions. The experiments were carried out 48 h after transfection.

### Lentivirus infection

The target fragment and packaging plasmid of human lentivirus shSeptin4 were purchased from Shanghai Genechem Co.,Ltd. (shSeptin4 target sequence 1: ccTAAAGGAAAGCATCCCATT; shSeptin4 target sequence 2: ccTAAAGGAAAGCATCCCATT; shSeptin4 target sequence 3: ccTAAAGGAAAGCATCCCATT). Lentivirus was produced in HEK-293T cells that were transfected with shRNA-expression vector. The virus supernatant was centrifuged at 1500 g for 5 min to remove cell fragments. The supernatant was then put into a chromatography cabinet for 24 h and centrifuged at 1500 g for 20 min at 4 °C. The virus particles were suspended in PBS and was added to the target cells. After 72 h of infection, 1 μg/ml puromycin was added for screening and cells were cultured for 7 days.

### Western blotting

The cell precipitate was collected and washed with precooled PBS, and then centrifuged at 1000 g for 5 min at 4 °C. Whole cells were lysed with lysis buffer containing 100 × protease inhibitor and 100 × PMSF and centrifuged at 13500 rpm at 4°C for 20 min. In general, 30-50 μg protein was added to 6 × protein loading buffer (final concentration, 1 ×) and boiled for 5-10 min before being subjected to SDS-PAGE and transferred onto PVDF membranes (Merck KGaA, Darmstadt, Germany). The membranes were incubated with blocking solution (TBST + 5% BSA) at room temperature for 1 h, then specific primary antibodies: goat polyclonal anti-Septin4 (ab166788, Abcam, UK), rabbit polyclonal anti-cleaved-caspase3 (19877-1-AP, Proteintech, USA), rabbit polyclonal anti-cleaved-PARP1 (5625S, Cell Signaling Technology, USA), rabbit polyclonal anti-BAX (50599-2-lg, Proteintech, USA), rabbit polyclonal anti-PCNA (10205-2-AP, Proteintech, USA), mouse monoclonal anti-Flag (GNI4110-FG, GNI, Japan), mouse monoclonal anti-GAPDH (10494-1-AP, Proteintech) or mouse monoclonal anti-β-actin (mAbcam 8226, Abcam, UK) were added and the blots were slowly shaken overnight at 4°C. The membranes were washed before incubation with the relevant secondary antibody at room temperature for 1 h. Blots were exposed to ECL luminescence reagent and images were collected using the chemiluminescence system (Tanon, TanonScience & Technology Co., Ltd., China).

### Immunoprecipitation

Cell precipitates were collected and lysed with IP lysis (137 mM NaCl, 10 mM NaF, 50 mM Tris HCl (ph 7.6), 1 mM EDTA, 0.1 mM Na_3_PO_4_, 10% glycerol, 1% NP-40, and 1 mM PMSF). Then 1 mg protein supernatant was incubated with the corresponding antibody for 3 h at 4 ℃ and placed with protein A/G beads (sc-2003, Santa Cruz, USA) for 12 h at 4 ℃. The beads were then washed three times with precooled lysate at 4 ℃ for 15 min, then subjected to SDS-PAGE.

### Immunohistochemistry

HCo1a180su17, a human colon cancer chip, was purchased from Outdo Biotech CO., Ltd (Shanghai, China). It contained 80 cases of cancer and adjacent tissues for which the survival information of patients was known. The colon cancer chip was treated as follows: 45 ℃ for 1 h, dried at 42 ℃ for 1 h, 72 ℃ for 3 h, and 2 h at 42 ℃ before staining. Xylene was used for dewaxing, and an ethanol gradient of anhydrous ethanol, 90%, 80%, and 70% ethanol was used for gradient dehydration for 5 min at each concentration. Then the chip was soaked in water for 5 min, and finally in PBS. Antigen retrieval was carried out with high pressure heating in sodium citrate buffer (pH 6.0) for 2 min. The chip was incubated with goat anti-Septin4 overnight at 4 °C. Finally, the chip was dehydrated in a 70%, 80%, and 90% ethanol gradient, followed by xylene dehydration. The chip was sealed with neutral resin, dried, and stored at room temperature, before imaging by optical microscopy.

### CCK8 cell viability assay

Cells were seeded in 96-well plates at a density of 3000 cells/well. After treatment, the cell culture medium was removed and replaced with 90 μL serum-free 1640 medium. Cell Counting Kit-8 (CCK8, Dojindo, Japan) staining solution (10 μL) was added to the cells and incubated at 37 ℃ for 2 h, then absorbance at 450 nm was measured by Absorbance Reader (Tecan, Switzerland).

### DHR reactive oxygen species staining

According to the required dosage, DHR probes (Sigma-Aldrich, St. Louis, MO) were diluted with probe diluent, mixed well, and stored in the dark. The final concentration of DHR was selected in the range of 100 times of dilution, the incubation time was selected as 2 h, and the incubation was conducted at room temperature in the dark. The cells were washed with PBS. Finally, the DHR was excited at 488 nm and the emission was measured at 525 nm by fluorescence microscopy.

### In vivo xenograft tumor growth

Female BALB/c nude mice of 4-5 weeks old were purchased from Beijing HFK Bioscience Co., Ltd. (Beijing, China). 1×10^7^ NC or shSeptin4 HCT116 stable cells were injected into the armpits of nude mice(n=6). Tumors had fully developed by day 4. Tumor size was measured every three days, and tumor volumes were calculated as length × width × height (mm^3^). On day 28, the mice were killed and photographed, and then the tumors were excised, weighed, and photographed.

### Statistical analysis

SPSS 22.0 software (SPSS Inc, Chicago IL, U.S.) was used for data analysis. The data are expressed as mean ± standard deviation (SD). Statistical significance was analyzed using a Student's *t*-test or a one-way analysis of variance followed by a Tukey's multiple comparisons test. Kaplan-Meier survival analysis was used to assess the association of Septin4 expression and CRC prognosis. *P*<0.05 represented statistical significance.

## Results

### Septin4 expression decrease with increased malignancy of colon cancer and are associated with survival outcomes

**Figure 1:**
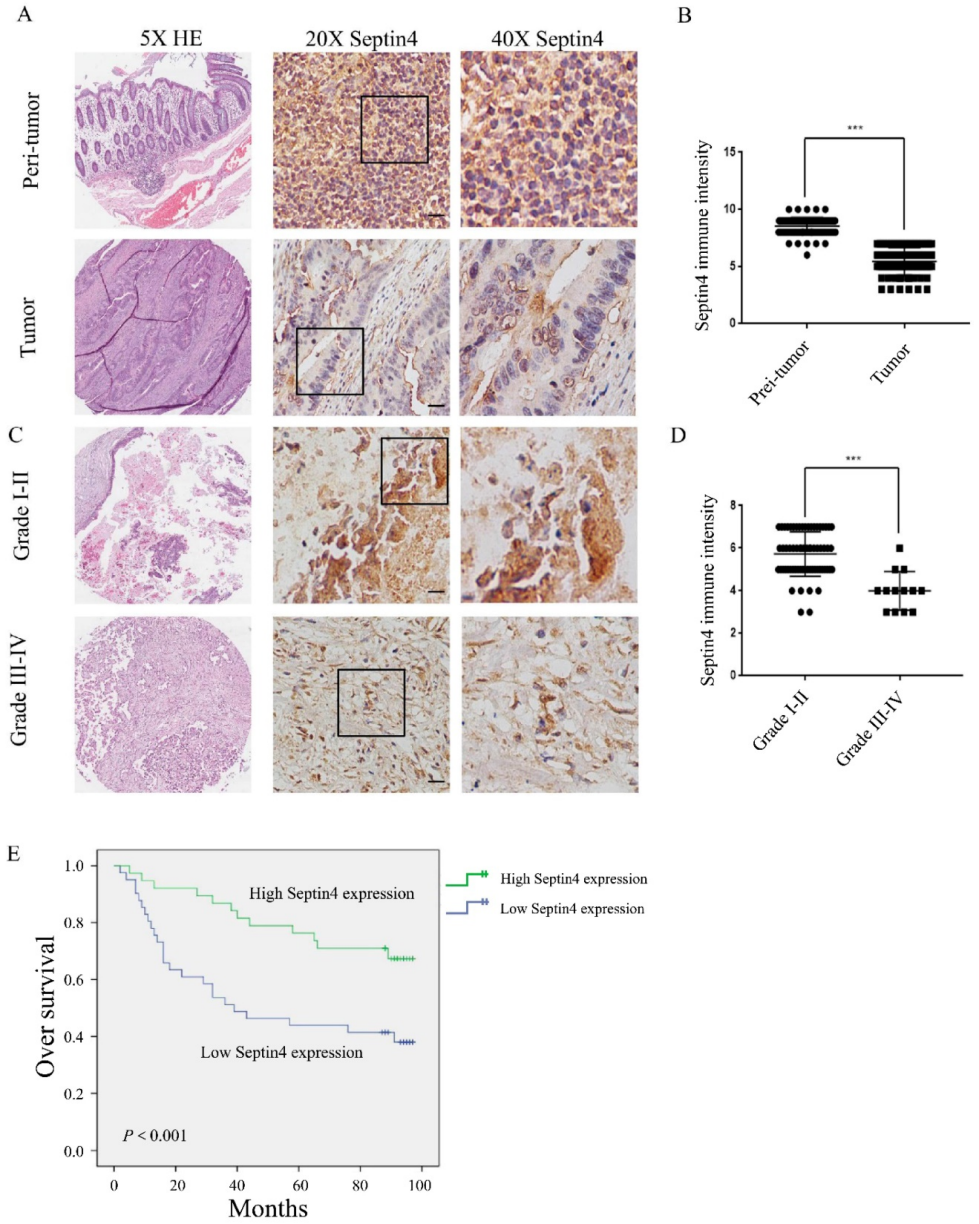
** Septin4 expression was correlated with good prognosis in human colorectal cancer. A.** Septin4 protein expression in CRC and paired peritumoral colonic tissue (n=79) was detected by immunohistochemical staining. Scale bars, 20 μm. **B**. Scores indicated Septin4 levels by a 10-point quantification scale, data are shown as means±S.D., ***P<0.001. **C.** Immunohistochemical staining of Septin4 was displayed in clinical CRC at different grades. Scale bars, 20 μm. **D**. Column plot analysis of Septin4 expression in CRC samples at different grades, data are shown as means±S.D., ***P<0.001. **E.** Kaplan-Meier survival curve showing high Septin4 expression (score 6-10, n=38) was associated with better overall survival compared with low Septin4 expression (score 0-5, n=41).

Firstly, we examined the expression of Septin4 in tissue specimens from 79 colon cancer patients, to elucidate the role of Septin4 *in vivo.* Septin4 expression and the patient clinicopathological parameters were analyzed. Table 1 shows that there was no significant difference between Septin4 expression and sex, age, histopathological TNM staging (*P*>0.05). However, based on grade, Septin4 expression was correlated with grade I-II and III-Ⅳ (*P*<0.001) (Table 1). Semiquantitative immunohistochemical analysis revealed a 1.6-fold higher level of Septin4 in peritumoral benign colon cancer cells when compared with colon cancer cells (Figure 1A, B; mean score 8 vs 5, *P*<0.001). Furthermore, Septin4 expression was significantly lower in advanced CRC cells (grade Ⅲ-Ⅳ) (Figure. 1C, D). Next, we examined whether or not Septin4 expression was associated with prognosis of CRC. Low Septin4 expression (score 0-5) was associated with a significantly worse overall survival when compared to those with a high expression level (score 6-10; Figure 1E). These results indicate that low Septin4 expression is implicated in the prognosis of CRC at advanced grade.

### Septin4 is involved in the apoptotic cell death of colon cancer cells

**Figure 2:**
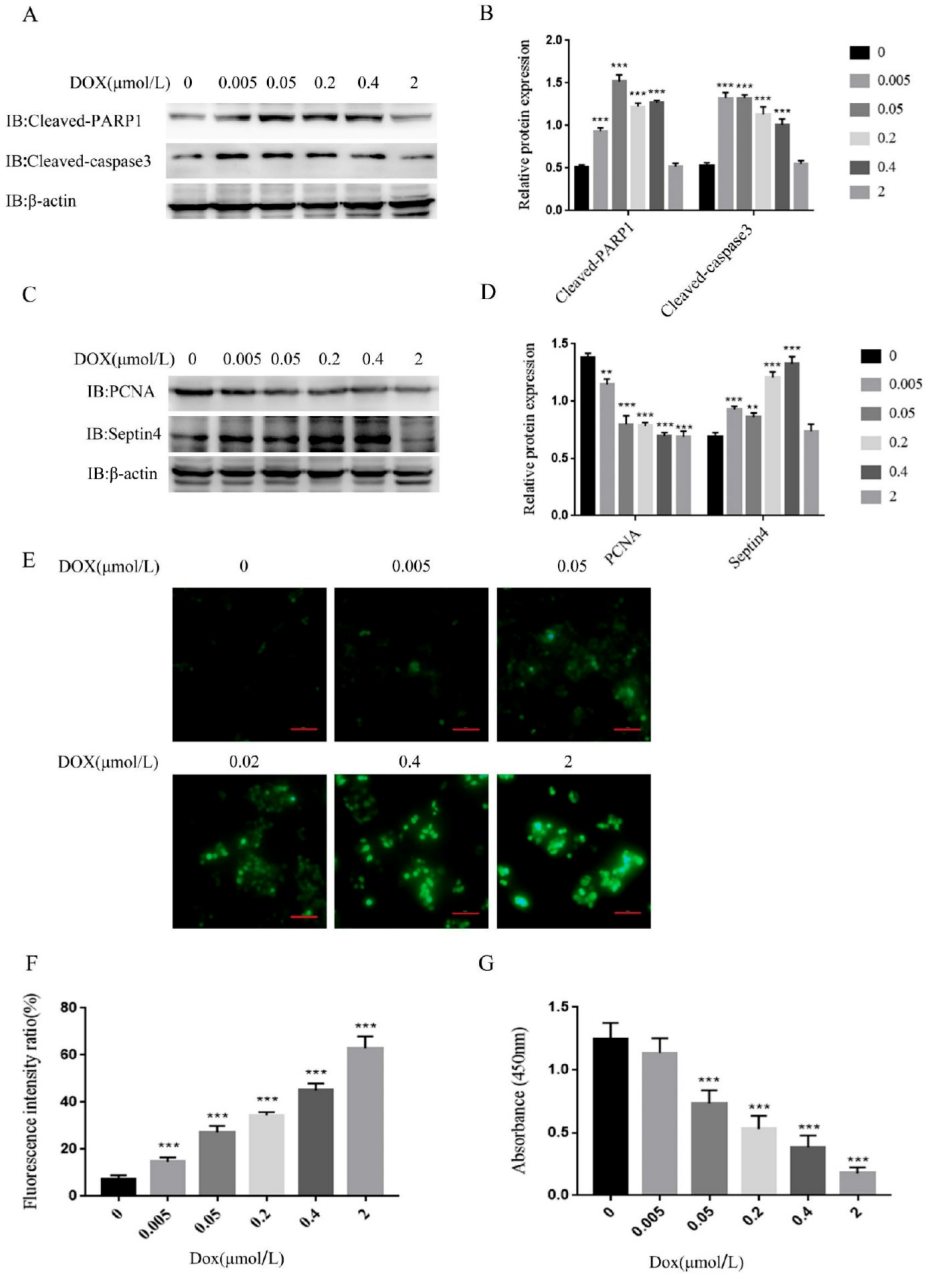
** Septin4 participated in DOX-induced apoptosis of HCT116 cells. A.** HCT116 cells were treated with DOX at 0, 0.005, 0.05, 0.2, 0.4, and 2 μmol/L for 48 h, to detect the expression of cleaved-caspase3 and cleaved-PARP1. B. Quantification of protein expression in A were shown as the means±S.D., ***P<0.001 C. The expression of PCNA and Septin4 was detected in the presence of DOX at 0, 0.005, 0.05, 0.2, 0.4, and 2 μmol/L for 48 h. D. Quantification of protein expression in C were shown as the means±S.D., **P<0.01, ***P<0.001. E. The generation of ROS was detected by DHR staining under the treatment of 0, 0.005, 0.05, 0.2, 0.4, and 2 μmol/L DOX for 48 h. Scale bars, 100 μm. F. Quantitative analyses of the fluorescence intensity in E, data are shown as means±S.D., ***P<0.001. G. Statistical results of CCK8 analysis of HCT116 treated with DOX at 0, 0.005, 0.05, 0.2, 0.4, and 2 μmol/L for 48 h, data are shown as means±S.D., ***P<0.001.

As it was revealed that Septin4 expression is correlated with grade and survival outcomes in CRC, we investigated the significance of Septin4 expression in CRC biology. The apoptosis of the human colon cancer cell line HCT116 was induced by a concentration gradient of DOX *in vitro*. The results showed that the apoptosis-related proteins, cleaved-PARP1 and cleaved-caspase3, were increased with the increase in DOX concentration, and reached a peak under the induction of 0.05 μmol/L DOX (Figure 2A, B). PCNA was also detected as an index to evaluate cell proliferation status. High level of PCNA is associated with poor prognosis in CRC patients and is a reliable and prognostic biomarker^25^-^27^. The expression of PCNA was decreased with increased DOX concentration, reaching the minimum at 0.05 μmol/L DOX, while Septin4 was increased with the increase in DOX and reached a peak at 0.4 μmol/L DOX (Figure 2C, D). Therefore, we determined that 0.05 μmol/L DOX was the best concentration to induce apoptosis of HCT116 cells. We also detected changes in PCNA and Septin4 mRNA expression under DOX treatment. As shown in Figure S1, PCNA mRNA was significantly reduced after DOX treatment for 48h, while Septin4 mRNA expression was increased. In addition, the changes in the protein level of cleaved-PARP1, cleaved-caspase3, PCNA and Septin4 was detected under time gradient conditions. PCNA expression was significantly reduced at 48 h, while cleaved-PARP1, cleaved-caspase3, and Septin4 were increased (Figure S2). These findings demonstrated that DOX could induce the apoptosis of HCT116 cells and that Septin4 was involved in this process. Then, DHR staining was performed to detect ROS production under a DOX concentration gradient. HCT116 induced by DOX at 2 μmol/L showed the highest ROS production (Figure 2E, F). Similar results were observed using CCK8 to analyze the viability of cells, and under 2 μmol/L DOX treatment cells were almost completely dead (Figure 2G). These results indicate that Septin4 is involved in the apoptosis process of HCT116 cells.

### Overexpression of Septin4 aggravated DOX-induced apoptosis of colon cancer cells

**Figure 3:**
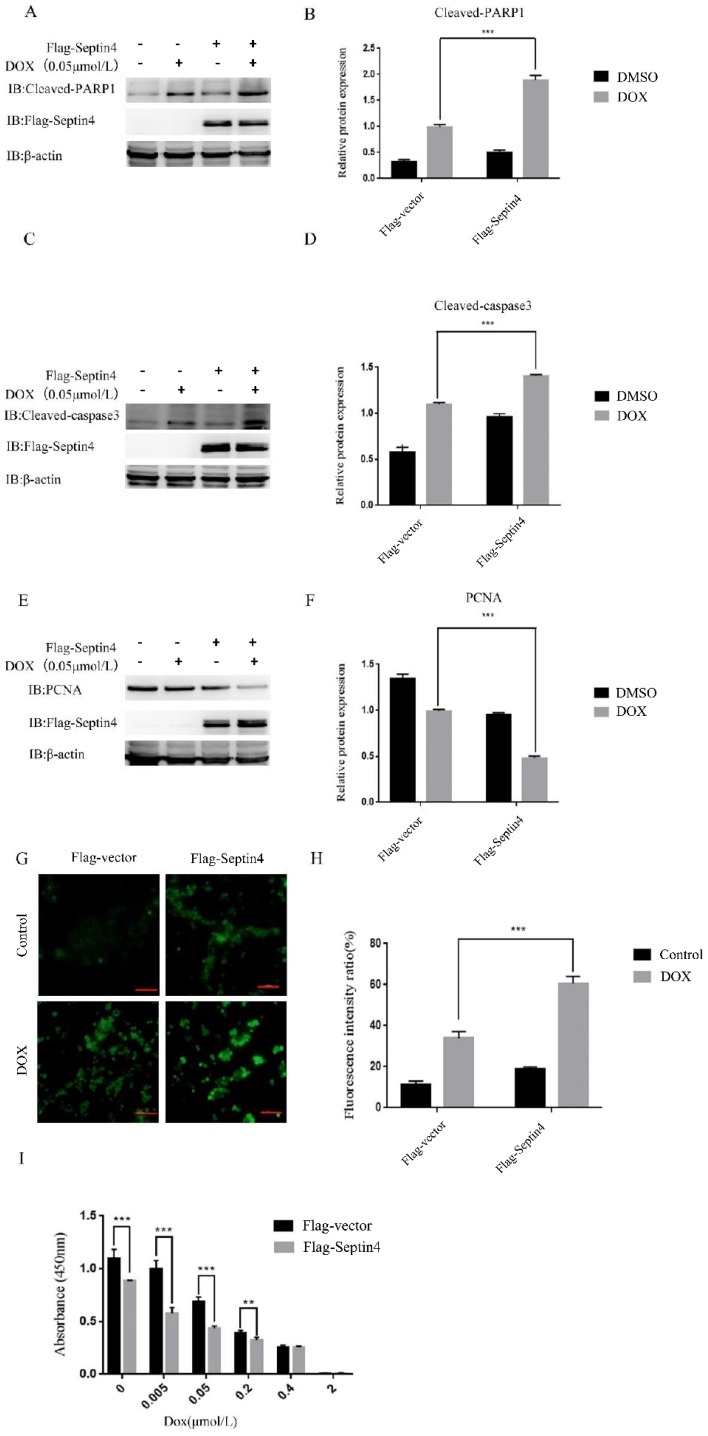
** Overexpression of Septin4 enhanced sensitivity to DOX in colon cancer cells. A.** HCT116 overexpressing Flag-Vector and Flag-Septin4 were treated with DOX at a concentration of 0.05 μmol/L for 48 h to detect the expression of cleaved-PARP1 and Flag-Septin4. **B.** Quantitative analysis of the expression of cleaved-PARP1 in **A**. Data were shown as the means±S.D., ***P<0.001. **C.** HCT116 overexpressing Flag-Vector and Flag-Septin4 were treated with DOX at a concentration of 0.05 μmol/L for 48 h to detect the expression of cleaved-caspase3 and Flag-Septin4. **D.** Quantitative analysis of the expression of cleaved-caspase3. Data were shown as the means±S.D., ***P<0.001.**E.** HCT116 overexpressing Flag-Vector and Flag-Septin4 were treated with DOX at a concentration of 0.05 μmol/L for 48 h to detect the expression of PCNA and Flag-Septin4. **F.** Quantitative analysis of the expression of PCNA in **E**. Data were shown as the means±S.D., ***P<0.001. **G.** The generation of ROS was detected by DHR staining in HCT116 overexpressing Flag-Vector and Flag-Septin4 treated with DOX at a concentration of 0.05 μmol/L for 48 h. Scale bars, 100 μm. **H.** Quantitative analyses of the fluorescence intensity in **G**. Data are shown as means±S.D., ***P<0.001. **I.** Statistical results of CCK8 assay of HCT116 overexpressing Flag-Vector and Flag-Septin4 under the DOX treatment at 0, 0.005, 0.05, 0.2, 0.4, and 2 μmol/L for 48 h. Data are shown as means±S.D., **P<0.01, ***P<0.001.

Next, we determined the role of Septin4 in DOX-induced apoptosis of colon cancer cells. We used normal colon cancer cells and Septin4-overexpressed colon cancer cells with or without 0.05 μmol/L DOX to induce apoptosis. We detected the expression levels of apoptosis-related proteins cleaved-PARP1 and cleaved-caspase3 by immunoblotting. The results showed that cleaved-PARP1 (Figure 3A) and cleaved-caspase3 (Figure 3C) increased significantly in the Septin4-overexpressed colon cancer cells induced by DOX compared with the control group. Also, the expression of PCNA in the Septin4-overexpressed cells decreased significantly compared to the control group under DOX stimulation (Figure 3E). Consistent with these findings, ROS production evaluated by DHR staining in figure 3G showed a significant increase in ROS production compared with the control group under DOX induction. These findings were consistent with apoptosis rate detected by TUNEL assay (Figure S3). CCK8 analysis also demonstrated that the cell viability decreased significantly after overexpression of Septin4 was induced by DOX (Figure 3I). In conclusion, overexpression of Septin4 aggravated DOX-induced apoptosis of colon cancer cells.

### Knockdown of Septin4 alleviated DOX-induced apoptosis of colon cancer cells

**Figure 4:**
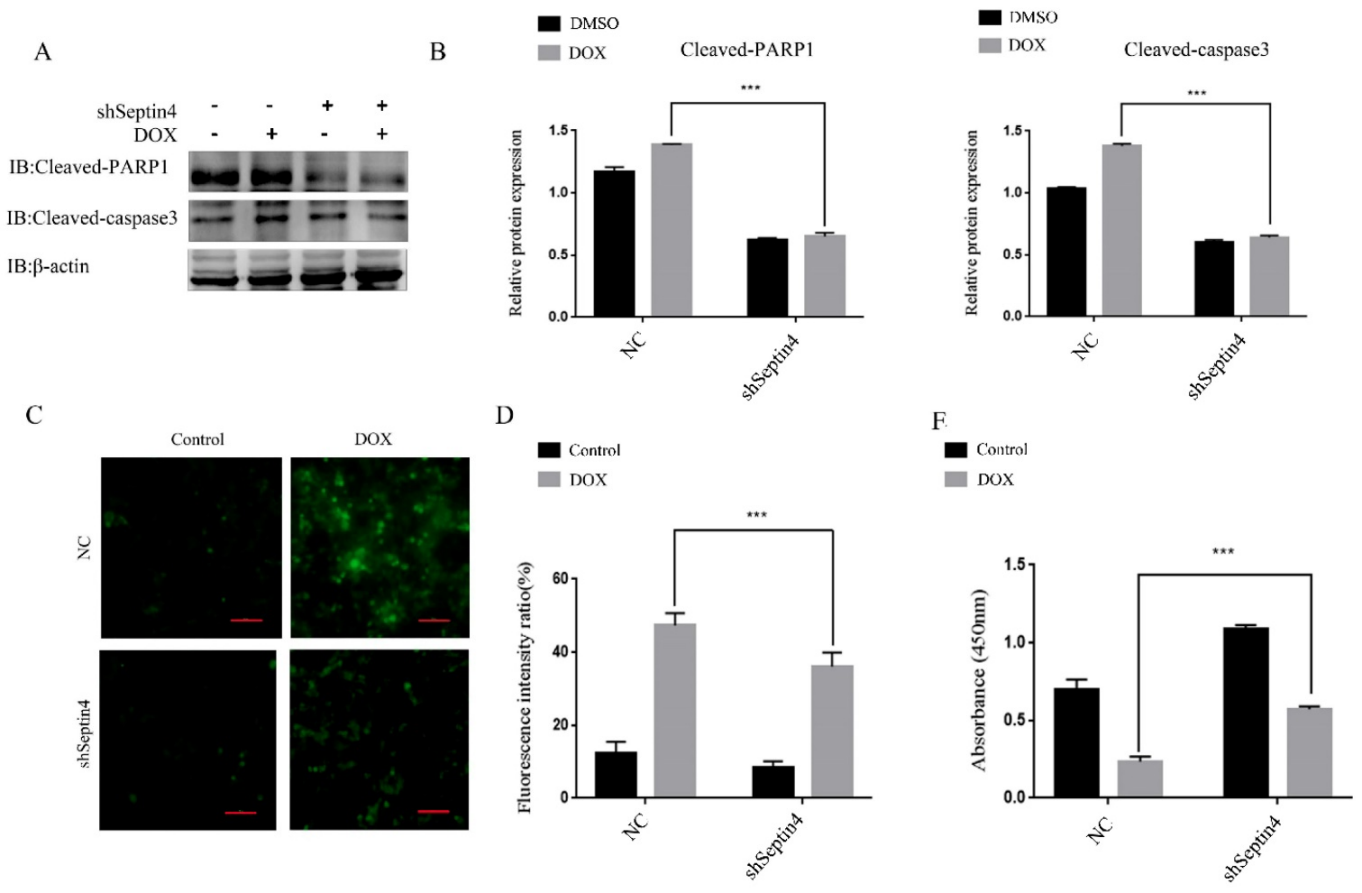
** Depletion of Septin4 resisted DOX-induced apoptosis in HCT116 cells. A.** Expression of cleaved-PARP1 and cleaved-caspase3 in NC and Septin4-knockdown HCT116 cells were treated with DOX at 0.05 μmol/L for 48 h. **B.** Quantitative analysis of the expression of cleaved-PARP1 and cleaved-caspase3 in **A**. Data were shown as the means±S.D., ***P<0.001. **C.** ROS staining of the NC and shSeptin4 HCT116 cells were treated with DOX at 0.05 μmol/L for 48 h. Scale bars, 100 μm. **D.** Quantitative analyses of the fluorescence intensity in **C**. Data are shown as means±S.D., ***P<0.001. **E.** Statistical results of CCK8 analysis of NC and shSeptin4 HCT116 cells under 0.05 μmol/L DOX treatment for 48 h, ***P<0.001.

To further determine the role of Septin4 in DOX-induced apoptosis of colon cancer cells, a stable knockdown of Septin4 HCT116 cell line was used to detect the expression levels of apoptosis-related proteins cleaved-PARP1 and cleaved-caspase3 by immunoblotting with and without DOX-induced apoptosis. We found that the expression of cleaved-PARP1 and cleaved-caspase3 was decreased significantly after knockdown of Septin4 compared to NC group under DOX treatment (Figure 4A). And in the shSeptin4 cells, we could not detect a significant increase of cleaved-PARP1 and cleaved-caspase3 after DOX treatment (Figure 4A). These results indicated that Septin4 is a key pro-apoptotic factor in DOX-induced CRC cell death. Then ROS staining was performed to evaluate the production of oxygen free radicals. The results showed that ROS production after knockdown of Septin4 decreased significantly compared to NC group under DOX treatment (Figure 4C). These findings were consistent with apoptosis rate detected by TUNEL assay (Figure S4). CCK8 analysis also showed that cell viability was significantly increased compared with the NC group under DOX treatment (Figure 4E). These results indicate that DOX-induced apoptosis is weaken by Septin4 depletion in CRC cells.

### Septin4 promoted apoptosis of colon cancer cells through interaction with BAX

**Figure 5:**
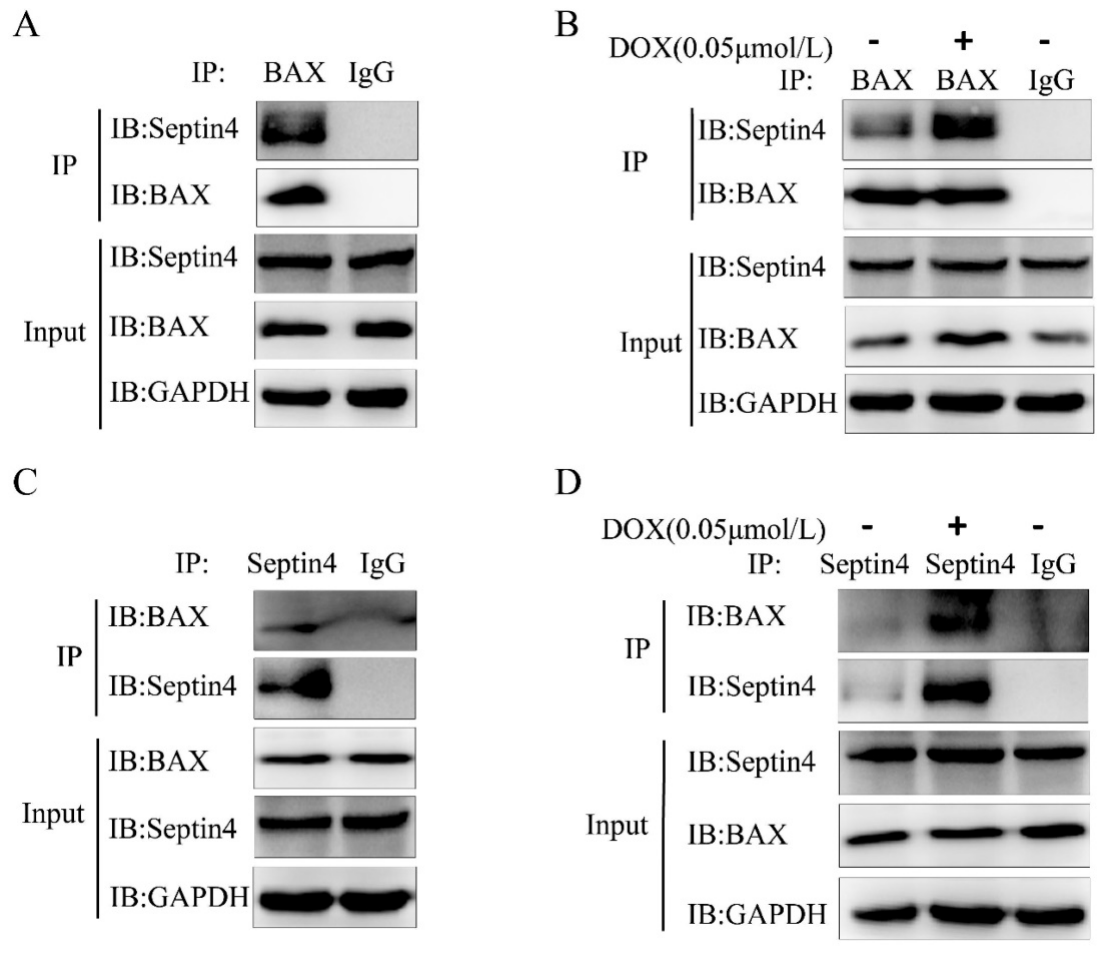
** Septin4 promoted apoptosis of colon cancer cells through interactions with BAX. A, C.** Immunoblotting with antibodies against Septin4 and BAX following immunoprecipitation using control species matched IgG and anti-BAX antibody (**A**) or anti-Septin4 antibody (**C**) in HCT116 cells. **B, D.** Immunoblotting with antibodies against Septin4 and BAX following immunoprecipitation using control species matched IgG and anti-BAX antibody (**B**) or anti-Septin4 antibody (**D**) in HCT116 cells with or without 0.05 μmol/L DOX treatment for 48 h.

We next explored which protein is regulated by Septin4 to promote the apoptosis of colon cancer cells induced by DOX. BAX is an apoptotic protein with multiple BH domains located in the outer membrane of mitochondria and has the ability to self-assemble into homologous oligomers to disrupt MOMP. BAX exists in almost all cell types, including tumor cells. The early model of apoptosis activation suggests that BAX activation is regulated by the inhibitory interaction of anti-apoptotic members on the outer mitochondrial membrane 24. We confirmed that BAX and Septin4 interacted with each other (Figure 5A, C) and that this interaction was enhanced under DOX induction (Figure 5B, D) by endogenous immunoprecipitation. These findings suggest that Septin4 can promote the apoptosis of colon cancer cells induced by DOX through interactions with BAX.

### Septin4 knockdown promotes human colorectal cancer growth *in vivo*

**Figure 6:**
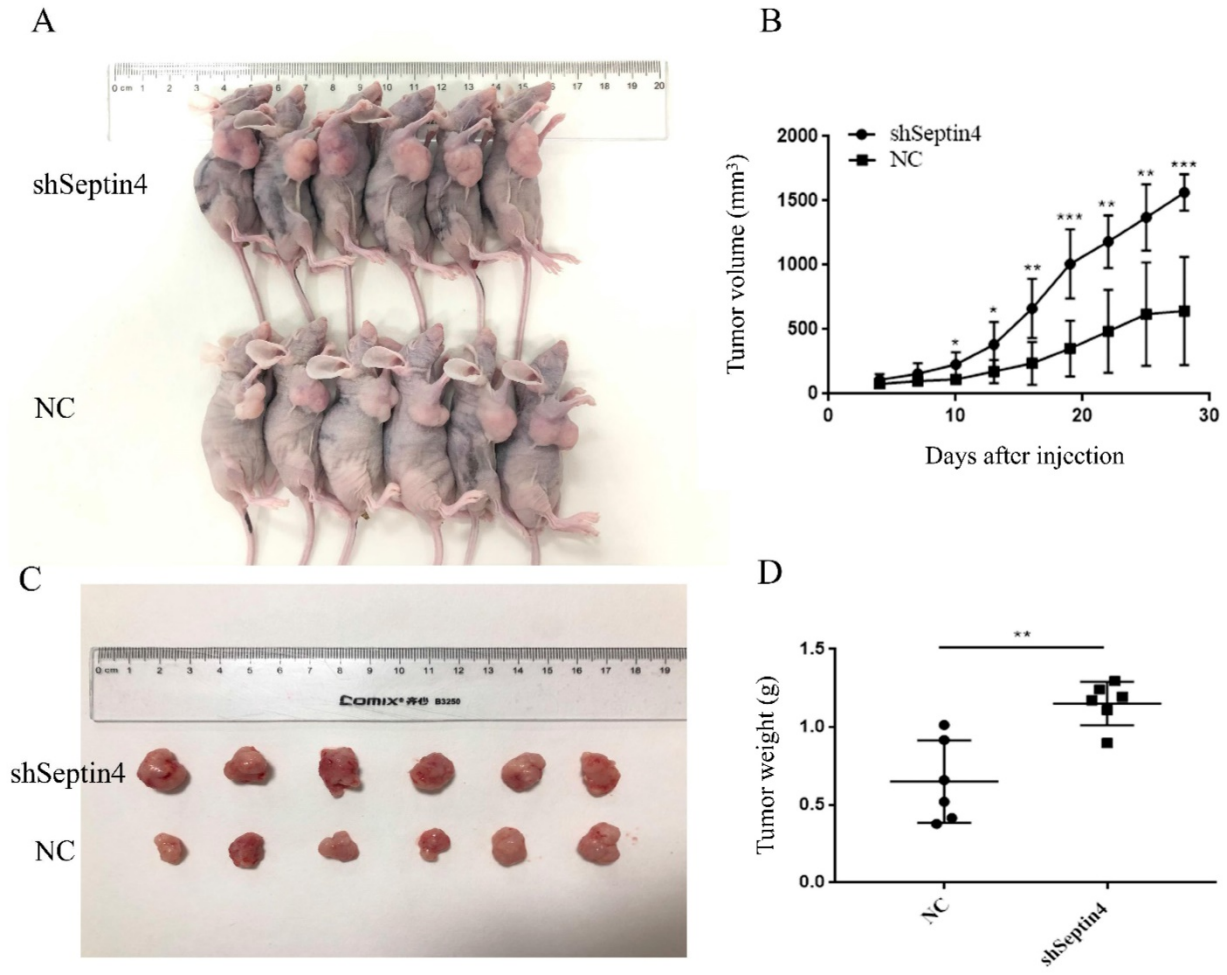
** Suppression of Septin4 enhances human colorectal cancer growth in vivo. A.** Xenografts was generated using HCT116 cells with stable Septin4 knockdown and normal control. Mice were killed and photographed at day 28. **B.** Tumor size was measured every 3 days and tumor volume was analyzed. Septin4-knockdown (shSeptin4) or control (NC) HCT116 cells were used in this analysis. The means±S.D. of tumor volume are shown. *P < 0.05; **P < 0.01, ***P < 0.001.** C.** Macroscopic images of transplanted tumors excised at day 28. **D.** The tumor weight was calculated on day 28. Data are shown as mean ± SD (n=6). **P<0.01.

To test whether Septin4-depleted tumors are accelerated growth, a mouse-tumor model was established using NC and shSeptin4 HCT116 cells. We subcutaneously injected shSeptin4 HCT116 cells and NC cells into nude mice and analyzed tumor growth. The Septin4 deficient HCT116 cells demonstrated larger tumor size than the normal control HCT116 cells (Figure 6A, B). In addition, Figure 6 B shows that cells with Septin4-knockdown had a greater tumor growth rate than normal control HCT116 cells. Furthermore, compared with normal control cells, Septin4*-*knockdown HCT116 cells also had a greater tumor weight (Figure 6C, D). Collectively, our data suggested that Septin4 knockdown promoted colon tumor growth *in vivo*.

## Discussion

In this study, for the first time, we confirmed that Septin4 expression levels were decreased with increased colon cancer grade, and patients with high expression of Septin4 had a good prognosis. We used Septin4-overexpressing and -knockdown cell lines to demonstrate Septin4 can promote the apoptosis of colon cancer cells and ROS production. Then we identified BAX as a new downstream substrate of Septin4 by immunoprecipitation. The results showed that Septin4 promoted apoptosis of colon cancer cells by binding to BAX. Then we performed tumor formation *in vivo*, which revealed that Septin4 knockdown accelerated subcutaneous tumor growth in nude mice. These findings may contribute to future research on the occurrence and development of CRC and provide new technical approaches for targeted treatment of CRC.

Septin4_i2 (ARTS) is an important apoptotic tumor suppressor protein encoded by *SEPT4* gene. ARTS is located in the outer membrane of mitochondria and translocate to the cytoplasm after receiving apoptosis-related stimulation. There it can inhibit caspases by mediating the degradation of XIAP to promote caspase activation and apoptosis 15, 28. Previous research has showed that the mice without *Sept4/ARTS* gene has significantly improved wound healing and hair follicle regeneration, revealing that ARTS can promote skin wound healing by regulating hair follicle stem cell apoptosis 29. In addition, the absence of *Sept4/ARTS* gene can also increase the number of hematopoietic stem cells and progenitor cells, increase XIAP levels, enhance apoptosis, and accelerate tumor development 18. It is worth noting that the expression of ARTS is often lost in human leukemia, indicating that ARTS it may have a role in tumor inhibition 19. Therefore, we speculate that in the development of colon cancer, Septin4 may also exhibit a similar effect of inhibiting tumor growth. It has been reported that *SEPT4* gene expression is decreased in up to 11 tumor types, including central nervous system tumors, breast cancer, cervical cancer, liver cancer, lung cancer, and melanoma. Moreover, the difference in Septin4 gene expression is related to tumor grade, stage, and prognosis. By comparing the immunostaining of colorectal cancer and its adjacent tissues, we found that Septin4 protein levels in tumor tissues were significantly lower than those in the adjacent tissues (Figure 1A, B), suggesting that the lack of Septin4 in colon cancer cells may be one of the critical factors in promoting the proliferation of cancer cells. This also suggested that Septin4 may act as a potential oncogene or tumor suppressor gene in the development of some cancer types. With colon cancer progression, we found that the expression of Septin4 decreased (Table 1, Figure 1C and D), which suggested that Septin4 has an important role in the inhibition of colon cancer development.

Some studies have shown that ARTS is deficient in all patients with acute lymphoblastic leukemia, and the degree of deficiency is significantly related to the malignant state of the disease. Leukemia cells lacking ARTS are resistant to cytosine arabinoside-induced apoptosis 19. However, after ARTS was transfected into these cells, they recovered their sensitivity to chemotherapy. To investigate whether Septin4 displays similar regulatory effects on the proliferation and apoptosis of colon cancer cells, we overexpressed Septin4 in HCT116 cells (Figure 3A). At the same time, we treated HCT116 cells with DOX, a DNA damaging agent that can inhibit proliferation and induce apoptosis by promoting the production of ROS. We examined the apoptosis-related proteins cleaved-PARP1 and cleaved-caspase3 by western blotting, ROS production, and cell viability by CCK8 assay, and confirmed that Septin4 promoted the process of apoptosis (Figure 3, S3). After DOX stimulation, the expression of cleaved-PARP1 and cleaved-caspase3 were increased, the production of ROS was increased significantly, and cell viability was decreased (Figure 3). Together, these results show that Septin4 can improve the sensitivity of HCT116 cells to DOX cytotoxic.

The location of Septin4 in the outer membrane of mitochondria is critical to its role in promoting apoptosis. The abnormal size of mitochondrion in spermatozoa with deletion of *Sept4* gene indicates the role of *Sept4* in mitosis and caspase-mediated cytoplasmic removal during spermatogenesis 30. In the mitochondrial apoptotic pathway, the release of apoptotic factors (including cytochrome *c* and Smac/Diablo (SMAC)) from the mitochondrial inter membrane space to the cytoplasm promotes caspase activation 23. After stimulation with apoptosis signals, Septin4 can transfer from the mitochondrial membrane to the cytoplasm, combine with XIAP, inhibit XIAP activity, and promote caspase activation to induce apoptosis 14. However, it is still unclear whether Septin4 can directly bind to mitochondrial membrane proteins to cause MOMP and promote the release of cytochrome *c*. Effective apoptosis requires BAX-/BAK-mediated increased MOMP 31. To activate MOMP, BH3 domain protein is required to bind and inhibit the pro-survival Bcl-2 family members, or directly bind and activate BAX and BAK, so as to increase MOMP 31, 32. As Septin4 shows a clear apoptotic effect in colon cancer cells (Figure 3 and 4), we speculate that mitochondrial membrane proteins may directly bind to it in addition to XIAP. Recent studies have reported that ARTS can combine with BCL-2 to form a XIAP-ARTS-Bcl-2 complex. Through the E3 ubiquitin ligase function of XIAP, the complex can mediate the degradation of Bcl-2 through the ubiquitin proteasome pathway to promote apoptosis 16. To clarify the mechanism of Septin4 in promoting the apoptosis of tumor cells, we found that Septin4 can interact with BAX and enhance this interaction following stimulation with DOX (Figure 5). This result revealed that Septin4 may increase MOMP, accelerate the release of cytochrome *c*, and promote apoptosis by directly combining with the mitochondrial membrane protein BAX. These results suggest that the interaction between Septin4 and BAX may have a critical role in inhibiting the development of colon cancer. This new mechanism of Septin4 provides a theoretical basis for its potential as a new target of colon cancer treatment. Finally, to verify these identified mechanisms *in vivo*, we subcutaneously injected HCT116 Septin4*-*knockdown or control cells into nude mice. The results showed that the volume of subcutaneous *in situ* tumors without Septin4 gene was significantly larger than those of the control group (Figure 6). Together, these findings indicated that Septin4 could inhibit the proliferation of tumor cells.

Surgery, chemotherapy, and radiotherapy are still the most common treatments for colorectal cancer 33. However, the effects of these traditional therapies are still not as good as expected. Therefore, it is imperative to identify a more effective treatment for colorectal cancer. Targeted therapies showing various advantages stand out among the current clinical treatment approaches for cancer and will be the focus of future drug development. Cetuximab and panitumumab, which are both monoclonal antibodies against EGFR, have been put into clinical application in the treatment of CRC 34, 35. EGFR is highly expressed in 60%-70% of CRC patients who can benefit from EGFR-targeted therapy 36, 37. However, these targeted cancer drugs are ineffective for patients with low EGFR expression or who have mutations in the extracellular domain of EGFR, and thus it is necessary to identify new therapeutic targets in addition to EGFR. Our study revealed for the first time that BAX, a new interacting protein of Septin4, has a critical role in mitochondrial apoptosis, suggesting that Septin4 may participate in controlling MOMP and promote apoptosis. However, this study also has some limitations. The correlation between Septin4 and CRC invasion and metastasis needs to be further clarified. In future experiments, we will further explore the role of Septin4 in CRC resistance.

In conclusion, we revealed low Septin4 expression in colon cancer. Overexpression of Septin4 can increase the chemosensitivity of colon cancer and inhibit its progression. In addition, Septin4 and BAX interact to regulate the apoptosis of colon cancer cells. These findings suggest that the Septin4/BAX pathway may be a new target of CRC treatment.

## Supplementary Material

Supplementary materials and methods, figures.

## Figures and Tables

**Table 1:** Relationship of Septin4 expression and clinicopathological parameters in colorectal cancer patients (n = 79)

	Septin4 immunostaining		
	Low (0-5)	High (6-10)	
Characteristic	N (41)	N (38)	*P*
Gender (N)			0.900
Male	20 (48.8)	18 (47.4)	
Female	21 (51.2)	20 (52.6)	
Age (years)	68.902±8.9883	66.947±10.9321	0.387
Grade			**0.001**
Ⅰ-Ⅱ	29 (70.7)	37 (97.4)	
Ⅲ, III-IV	12 (29.3)	1 (2.6)	
Clinical stages			0.190
Ⅰ-Ⅱ	21 (51.2)	25 (65.8)	
III-IV	20 (48.8)	13 (34.2)	
Lymph node metasis			0.179
N0	22 (53.7)	26 (68.4)	
N1	19 (46.3)	12 (31.6)	
Distant metasis			
M0	41 (100.0)	38 (100.0)	
M1	0 (0)	0 (0)	
