# Supplementary material for: Septin4 promotes cell death in human colon cancer cells by interacting with BAX
Source: Int J Biol Sci. 2020 Apr 7;16(11):1917–28. doi: 10.7150/ijbs.44429 (PMC7211164; doi:10.7150/ijbs.44429)

Supplementary Materials for

Septin4 promotes cell death in human colon cancer cells by interacting with BAX

Supplementary Materials and Methods

Real-time quantitative polymerase chain reaction (real-time qPCR)

Total RNA of HCT116 cells after DOX treatment was extracted using RNAiso Plus (9109, Takara Bio, Japan) based on manufacturer's protocol, and total RNA was reverse transcribed using a PrimeScript™ RT reagent Kit (RR047A, Takara Bio, Japan) for RT-PCR (Roche). The real-time qPCR was performed with TB Green® Premix Ex Taq™ II Kit (RR820A, Takara Bio, Japan) using a LightCycler 96 (Roche). Primer sequences are shown as follows.

PCNA: 5'- TAA TTT CCT GTG CAA AAG ACG G -3', and 5'- AAG AAG TTC AGG TAC CTC AGT G-3'

Septin4: 5'- ACA CAT TAT GAG AAC TAC CGG G -3', and 5'- TTC TCA GTT TCT GGA TCT GTC C -3'

β-actin (as a control): 5'- CGA CAA CGG CTC CGG CAT GT-3', and 5'- CTT GCT CTG GGC CTC GTC GC-3'

TUNEL assay

The transfected Septin4-overexpressing HCT116 cells or knockdown cells were seeded into 24-well plates with a glass coverslip in each well. After DOX treatment, the cells were rinsed twice with PBS, fixed in 4% paraformaldehyde for 15 min at room temperature, and permeabilized with 0.1% Triton X-100. Apoptosis cells were

detected using a TUNEL BrightGreen Apoptosis Detection Kit (A112, Vazyme, China) according to manufacturer's protocol. Cells were observed using the NIKON Ti-S fluorescence microscope.

Supplementary Figures and Legends

Figure S1: PCNA and Septin4 mRNA changes under time course of DOX treatment.

HCT116 cells were treated with 0.05 μmol/L DOX at indicated time points, the transcription of PCNA and Septin4 is shown below. Data were shown as the means±S.D., ***P*<0.01, ****P*<0.001.

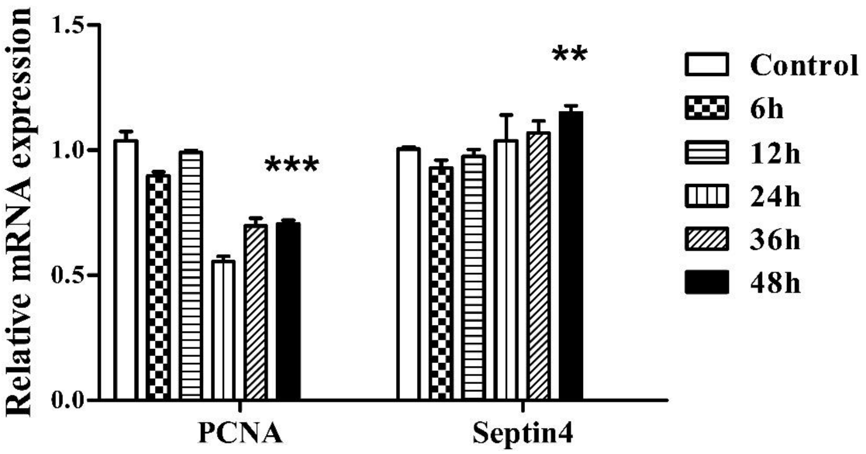

Figure S2: PCNA and Septin4 protein level changes under time course of DOX DOX-induced apoptosis.

HCT116 cells were treated with 0.05 μmol/L DOX at indicated time points, the expression of cleaved-PARP1, cleaved-caspase3, PCNA and Septin4 was detected by western blot.

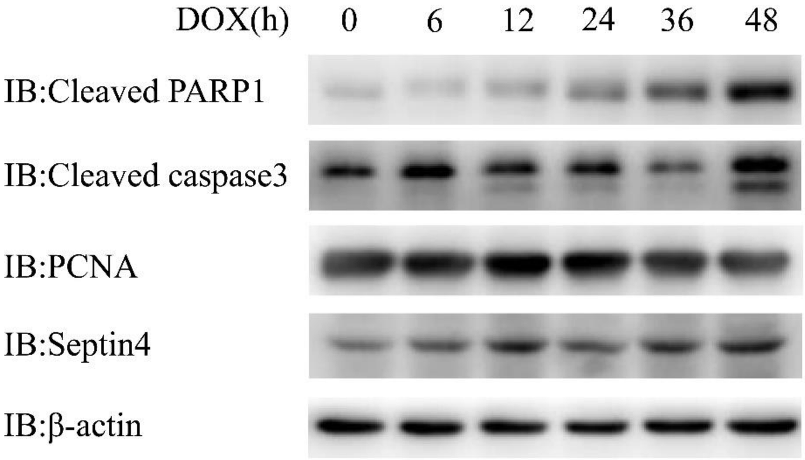

Figure S3: Overexpression of Septin4 enhanced apoptosis under DOX treatment in colon cancer cells.

The apoptosis cells were detected by TUNEL assay in HCT116 overexpressing Flag-Vector and Flag-Septin4 treated with DOX at a concentration of 0.05 μmol/L for 48 h. Scale bar, 200 μm. Quantitative analyses of TUNEL positive cells are shown as means±S.D., ***P<0.001.

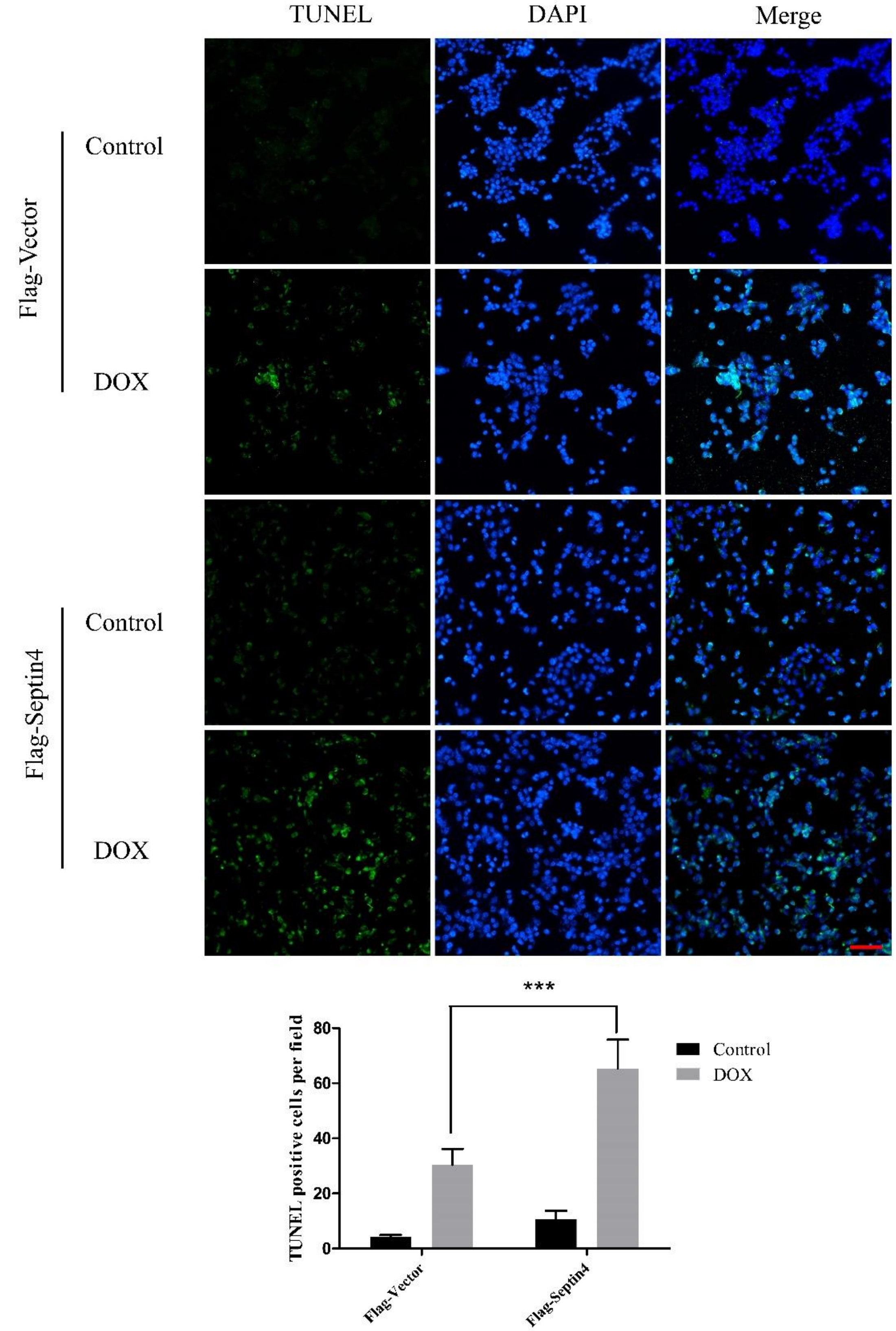

Figure S4: Depletion of Septin4 resisted DOX-induced apoptosis in HCT116 cells. The apoptosis cells were detected by TUNEL assay in NC and Septin4-knockdown HCT116 cells treated with DOX at a concentration of 0.05 μmol/L for 48 h. Scale bar, 200 μm. Quantitative analyses of TUNEL positive cells are shown as means±S.D., ***P<0.001.

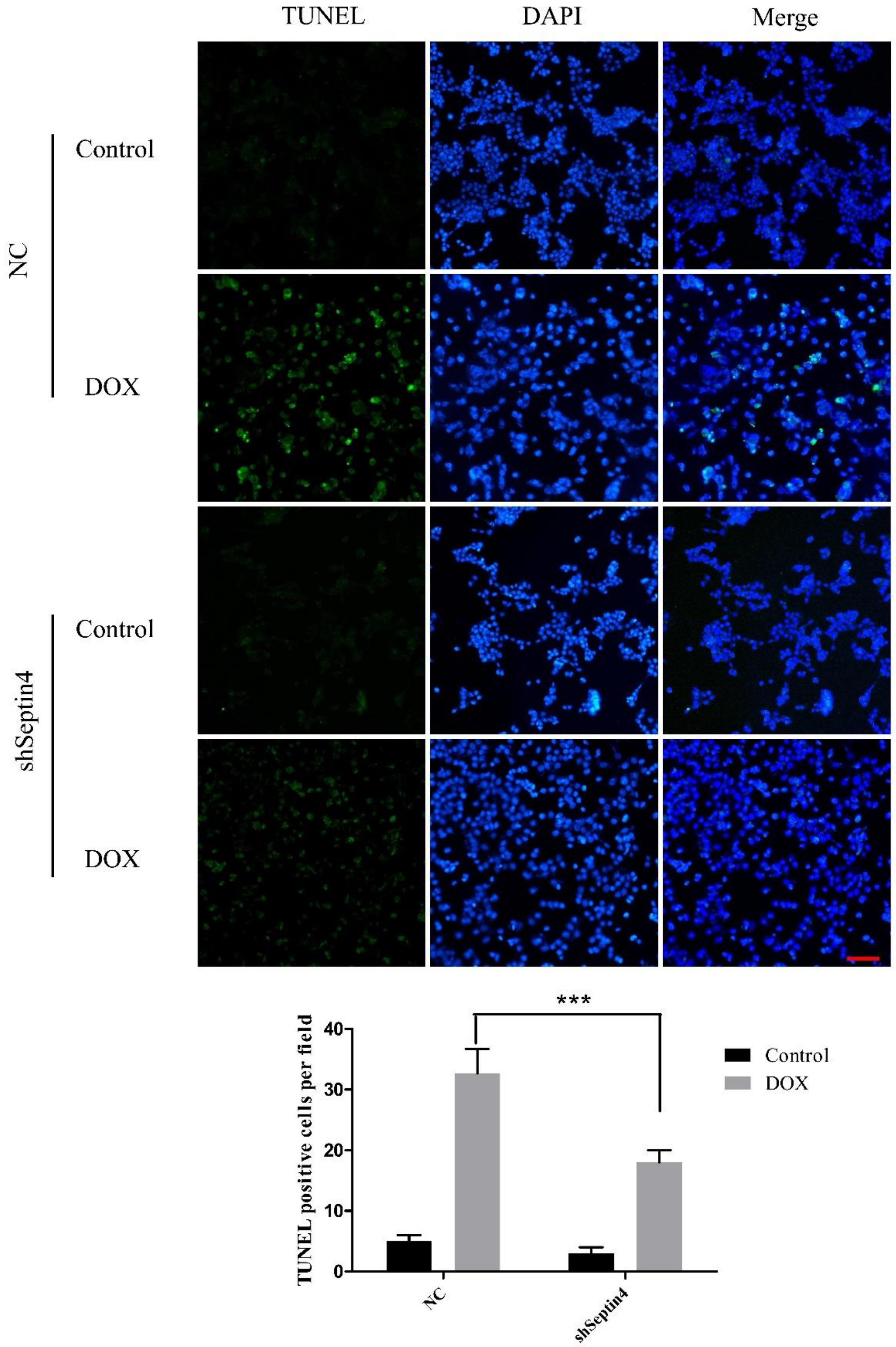

Supplement: Supplementary file 1 — Supplementary materials and methods, figures. [file ijbsv16p1917s1.pdf]
